# Allergic fetal priming leads to developmental, behavioral and neurobiological changes in mice

**DOI:** 10.1038/tp.2015.40

**Published:** 2015-04-07

**Authors:** J J Schwartzer, M Careaga, C Chang, C E Onore, P Ashwood

**Affiliations:** 1 Program in Neuroscience and Behavior, Department of Psychology and Education, Mount Holyoke College, South Hadley, MA, USA; 2 Department of Medical Microbiology and Immunology, University of California, Davis, Davis, CA, USA

## Abstract

The state of the mother's immune system during pregnancy has an important role in fetal development and disruptions in the balance of this system are associated with a range of neurologic, neuropsychiatric and neurodevelopmental disorders. Epidemiological and clinical reports reveal various clues that suggest a possible association between developmental neuropsychiatric disorders and family history of immune system dysfunction. Over the past three decades, analogous increases have been reported in both the incidence of neurodevelopmental disorders and immune-related disorders, particularly allergy and asthma, raising the question of whether allergic asthma and characteristics of various neurodevelopmental disorders share common causal links. We used a mouse model of maternal allergic asthma to test this novel hypothesis that early fetal priming with an allergenic exposure during gestation produces behavioral deficits in offspring. Mothers were primed with an exposure to ovalbumin (OVA) before pregnancy, then exposed to either aerosolized OVA or vehicle during gestation. Both male and female mice born to mothers exposed to aerosolized OVA during gestation exhibited altered developmental trajectories in weight and length, decreased sociability and increased marble-burying behavior. Moreover, offspring of OVA-exposed mothers were observed to have increased serotonin transporter protein levels in the cortex. These data demonstrate that behavioral and neurobiological effects can be elicited following early fetal priming with maternal allergic asthma and provide support that maternal allergic asthma may, in some cases, be a contributing factor to neurodevelopmental disorders.

## Introduction

Developmental neuropsychiatric disorders are heterogeneous in their presentation and often share common behavioral characteristics across diagnoses. For example, social behavior deficits, including difficulties relating to others, impaired mood regulation and cognitive impairments are apparent in autism spectrum disorders (ASD), schizophrenia, attention deficit/hyperactivity disorder (ADHD) and bipolar disorder. These shared characteristics represent plausible endophenotypes that may result from a common etiological mechanism. Although bipolar disorder and schizophrenia emerge later in life, these disorders along with other neurodevelopmental disorders, such as ASD and ADHD, are thought to have developmental origins early in the prenatal period.^1, 2, 3^ Although genetic factors are likely contributors to these disorders, heritability estimates indicate strong environmental contributions.^4^ Of particular interest is the link between fetal gestation and the activation of the maternal immune system during critical periods of development. Epidemiological reports suggest a strong association between periods of maternal immune activation and an increased risk of having a child with a neurodevelopmental disorder.^5, 6, 7^ This association is concerning given the parallel increases reported in the incidence of immune-related disorders, in particular asthma and allergies, over the past decade.^8, 9, 10, 11^

Much of the clinical and preclinical research has focused on the consequences of maternal pathogen exposure, such as bacteria and viruses, on fetal development. Pro-inflammatory factors released in response to viral or bacterial infections have been associated with schizophrenia, ASD, ADHD and bipolar disorder,^7, 12, 13, 14^ highlighting the immune response, not the specific pathogenic agent, as a contributing factor to the etiology of these diseases.^15^ However, unique immune cascades may be evoked in response to other immunogenic agents that are not of viral or bacterial origin. For example, allergies and asthma represent an alternative inflammatory response mediated by a T-helper type 2 (T_H_2) polarization resulting in humoral responses. Although both viral/bacterial and allergen-induced stimuli result in activation of the maternal immune system, different cytokine profiles and/or signaling cascades may contribute to unique consequences on fetal brain development. To date, little is known about the developmental consequences of maternal T_H_2-mediated responses, such as the effects of allergy/asthma induction, on fetal development. This is particularly important given the rises in allergies observed^16^ and the recent epidemiological links found between maternal allergies and risk of neurodevelopmental disorders.^17, 18^ These associations warrant a need to examine the neuroimmunological effects of maternal allergies and asthma on offspring development and behavior.

To date, preclinical investigations of maternal immune activation have relied exclusively on the use of viral and bacterial agents, most notably polyinosinic-polycytidylic acid [poly(I:C)] and lipopolysaccharide, to identify behavioral consequences of systemic prenatal infection.^15, 19^ Many of these reports have observed changes in various behaviors analogous to the common behavioral characteristics present across neurodevelopmental disorders including altered social behavior,^20, 21^ motor activity,^22^ anxiety^23^ and learning and memory impairments.^22, 23^ Although these findings demonstrate a link between maternal immune activation and neurodevelopmental deficits, the translational validity of pathogen-induced fetal priming is limited as rates of pathogen infection in developed countries have markedly declined over the past century.^24^ Conversely, incidence of neurodevelopmental disorders, such as ASD, are increasing at an alarming rate.^25^ This inconsistency suggests that maternal viral/bacterial infection is unlikely the cause of all cases of neurodevelopmental disorders and raises the question whether other immunogenic agents, particularly those stimuli that are currently on the rise, such as allergy and asthma, equally impact neurodevelopmental and neuropsychiatric disease prevalence.

The immune and nervous system are intimately connected throughout development as perturbations in one system impart developmental changes in the other.^26^ Among the numerous signaling pathways important for brain development, the serotonergic system emerges early in brain development, even before the establishment of conventional synapses, and has a vital role in neuronal proliferation, migration and synapse formation.^27, 28^ Moreover, accumulating evidence suggests that modification of the serotonergic system contributes to dysfunctional brain development associated with numerous neurodevelopment disorders. The serotonin transporter (SERT) has a central role in the regulation of serotonin signaling and pharmacological targets at this site, including the widely used selective serotonin reuptake inhibitors, are among the most popular treatments for several neurodevelopmental and neuropsychiatric disorders. Importantly in mice, SERT is present as early as embryonic day 8, a period when placental serotonin is believed to impart substantial influence on forebrain development^29, 30^ and expression of serotonergic proteins like SERT are regulated in response to immune system factors. This intimate connection between immune signaling cascades and brain development place the serotonin system, particularly SERT, at the apex of immune-related mental health and warrant investigation of how maternal exposure to allergy/asthma during pregnancy alters brain development, starting with changes in SERT forebrain expression.

We describe the first set of studies that tested the hypothesis that maternal allergy/asthma (MAA) imparts neurobehavioral alterations in brain and behavior of offspring reminiscent of the common endophenotypes observed in neurodevelopmental disorders. To test this, pregnant dams were induced with allergic airway inflammation and their offspring were measured for developmental and behavioral alterations to identify changes in sociability (social approach and dominance), repetitive and perseverative behaviors (grooming and marble burying), and hyper/hypoactivity (locomotor activity). In addition, offspring were assessed for general anxiety (elevated plus maze) and basic learning and memory behavior (novel object recognition) to identify whether differences in arousal states or cognitive ability contributed to behavioral changes. In addition, to begin to address potential mechanisms, brains of offspring born to MAA dams were examined for evidence of altered SERT expression following behavioral analysis.

## Materials and methods

### Animals

Male and female C57Bl/6J mice generated from breeding pairs purchased from Jackson Laboratory (Sacramento, CA, USA) were bred and maintained by the Center for Laboratory Animal Research, at the University of California, Davis, and maintained at ambient room temperature on a 12 h light/dark cycle (lights on at 0600 h). In addition, 129/SvImJ mice (Jackson Laboratory) were used as stimulus mice for the social approach task. Mice were group housed in standard plastic cages with same-sex litter mates, unless otherwise noted. Cages were maintained in a temperature- (20 °C) and humidity (55%)-controlled vivarium with food and water provided *ad libitum*. All behavioral tasks were performed during the first 4 h of the light cycle, and all procedures were performed with approval by the University of California, Davis Institutional Animal Care and Use Committee and in accordance with the guidelines provided by the National Institutes of Health Guide for the Care and Use of Laboratory Animals.

### Maternal allergy/asthma induction

Sexually naive female C57Bl/6J mice were sensitized with a single intraperitoneal injection of 10 μg ovalbumin (OVA, Sigma, St Louis, MO, USA) in 1 mg (Al)OH_3_ (InvivoGen, San Diego, CA, USA) dissolved in 200 μl phosphate-buffered saline (PBS) on postnatal day (P)42 and again 1 week later at P49. One week following the second sensitization treatment, female mice were mated overnight and checked daily for the presence of a seminal plug, noted as gestational day 0.5. Pregnant mice were randomly assigned to either the allergic asthma or control group and exposed to either an aerosolized solution of 1% (wt/vl) OVA in PBS (that is, the MAA group) or PBS control for three 45-minute induction sessions throughout gestation. Specifically, these induction sessions occurred at gestational days 9.5, 12.5 and 17.5, to correspond with early, middle and late gestation, as previously described.^21, 31, 32, 33^ Following the final induction, mice were returned to their home cages, single housed and left undisturbed until the birth of their litters. Pups remained with their mother until weaning on P21, at which time the offspring were group housed with same-sex littermates. A total of 23 dams (11 PBS and 12 MAA) were used to generate 82 offsprings (39 PBS, 43 MAA).

### Experimental design

Experimental offspring were separated into two cohorts to reduce the total number of behavioral tasks imparted on any one animal. For cohort 1, juvenile offspring were weighed and body length measured from P4 until P16 to identify difference in developmental trajectories. After weaning, these mice were tested for social approach behavior at 8 weeks of age followed by grooming and marble-burying tasks at 9 weeks of age. At 10 weeks old, mice in cohort 1 were measured for locomotor activity in the open-field arena followed by memory performance in the novel object recognition task. In cohort 2, 8-week-old mice were first tested for anxiety-like behavior in the elevated plus maze task to mitigate the potential anxiogenic effects of subsequent behavioral testing. One week following the elevated plus maze task, these mice were tested for social behavior deficits in the social approach task. Finally, at 10 weeks of age, mice in cohort 2 were tested for grooming and marble-burying behavior.

### Social approach

Changes in social approach behavior were measured between offspring of MAA and PBS-exposed dams using the three-chambered social approach task as previously described.^33, 34, 35^ Mice were placed in the center chamber and allowed to habituate for 10 min followed by an additional 10 min of free exploration of all three chambers to confirm the absence of a side preference. Then, experimental animals were returned to the center chamber while a sex-matched novel 129/SvImJ mouse was placed under an inverted wire cup in one side chamber, and an identical empty wire cup was placed on the other side. Mice were then given an additional 10-min period to explore both chambers and the time spent in each chamber was measured. The chamber containing the novel mouse was counter balanced to control for any bias in chamber preference. A sociability score was calculated as the time spent in the novel mouse chamber minus time spent in the novel-object chamber.

### Social dominance tube test

Social anxiety was evaluated on the basis of differences in social dominance using methods adapted from Spencer *et al.*^36^ One offspring of a MAA dam and one offspring of a PBS-exposed dam were placed head first at opposite ends of a 30-cm long acrylic tube (2.5-cm diameter) and released simultaneously. After meeting in the center, the less dominant mouse backed out leaving the ‘winning' mouse remaining in the tube. Each mouse was tested four times, each time with a novel same-sex opponent from the opposing treatment group, and the entry side was counter balanced across trials. For each trial, the winning mouse was granted a score of 1 and the submissive mouse earned 0. Matches lasting for more than 2 min with neither animal backing out ended in a draw, and each mouse received a score of 0.5.

### Marble burying

One week following the social approach task, mice were habituated for 10 min to a clean Plexiglas cage (37 × 14 × 12.5 cm) filled with a 4-cm thick layer of clean corncob bedding. Following habituation, animals were returned to their home cage and 15 glass marbles were laid out in five rows of three marbles placed equidistance apart. Mice were then returned to their cages and allowed to explore under dim illumination for 10 min. At the end of the 10-min period, animals were gently removed from the testing cages and the number of marbles buried was recorded. Only marbles covered by 75% or more bedding were counted as buried, as previously described.^21, 33^

### Grooming

Two days after marble burying, animals were placed in an empty clean Plexiglas cage and left undisturbed to habituate for 10 min. Following habituation, mice were video recorded for 10 min and later scored for self-grooming behavior by two individuals blind to treatment conditions. Grooming was defined as time spent licking paws, washing the nose and face, or scratching fur with any foot.

### Locomotor activity

Experimental animals were individually placed in an empty Plexiglas arena (30 cm × 30 cm × 38 cm) and allowed to freely explore the environment for 10 min. Horizontal locomotor activity was assessed using Ethovision XT 9 (Noldus Information Technology, Leesburg, VA, USA) video tracking software to measure total distance traveled and average velocity during the 10-min exploration task.

### Novel-object recognition

Memory performance was assessed in the open-field arena beginning with a 10-minute habituation period. The following day, mice were returned to the cleaned arena and allowed to freely explore two identical objects for a 10-minute familiarization phase. Twenty-four hours later, one of the familiar objects was replaced with a novel object and experimental mice were returned to the arena and video recorded for a 5-min testing session. Object investigation, as defined by the time spent sniffing either the familiar or novel object, was measured by two trained observers. Novel-object recognition was calculated by measuring the time spent sniffing the novel object divided by the total time sniffing either object. Novel-object sniff times significantly greater than 50% indicate object recognition. All objects and arenas were cleaned between each testing session with 70% ethanol to remove olfactory cues.

### Elevated plus maze

The apparatus was constructed of dark gray Plexiglas and consisted of two open arms (30 cm × 5 cm × 0.25 cm) and two perpendicular closed arms (30 cm × 5 cm × 15 cm) extending from a central platform. The entire apparatus was elevated ~1 m above the floor. At the start of testing, mice were placed on the center platform and allowed to freely explore the maze for 5 min. Behavioral responses were video recorded and later measured for the number of entries into each arm, as defined by all four paws in the arm, as well as the time spent in the open and closed portions of the maze. The percent of time spent exploring the open arms was calculated by the time in the open arm divided by the total time spent in either the open or closed arms. Reductions in open arm exploration were interpreted as increased anxiety.

### Immunoblot

One week following behavioral testing, one male and one female mouse from each litter were deeply anesthetized with isoflurane (4% inhalation) and brains removed for immunoblot analysis. Dissected cortex was homogenized in ice-cold lysis buffer containing 20  mM Tris-HCl (pH 7.5), 150 mM NaCl, 1 mM Na2EDTA, 1 mM EGTA, 1% NP-40, 1% sodium deoxycholate, 2.5 mM sodium pyrophosphate, 1 mM β-glycerophosphate, 1 mM Na_3_VO_4_ and 1 μg ml^−1^ leupeptin (Cell Signaling, Danvers, MA, USA). Lysates were then sonicated for 30 s and vortexed for 30 s before being centrifuged at 20 000 *g* for 10 min. Supernatants were collected and total protein was quantified by Bradford assay (Thermo-Fisher, Rockford, IL, USA). Twenty micrograms total protein were loaded onto a 4–20% Criterion XT precast gel (Bio-Rad, Hercules, CA, USA) per sample and electrophoresed at 100 V. The separated proteins were then transferred to a nitrocellulose membrane (Bio-Rad) at 100 V. After transfer, the membrane was blocked in 5% milk in Tris-buffered saline (TBS) for 1 h at room temperature. The membrane was then probed overnight at 4 °C in TBS with 0.05% Tween-20 (TBST; Sigma) using a dilution of 1:250 goat anti-SERT (Santa Cruz Biotechnology) and 1:5000 rabbit anti-β-Actin (Cell Signaling) antibodies. This was followed by incubation with donkey anti-goat conjugated to horseradish peroxidase (JacksonImmuno, West Grove, PA, USA) and donkey anti-rabbit conjugated to horseradish peroxidase both at 1:10 000 in TBST for 1 h at room temperature. Bands were then visualized using SuperSignal West Dura Chemiluminescent Substrate (Thermo-Fisher) using an Alpha Innotech FluorChem 8800 Image Detection System (ProteinSimple, Santa Clara, CA, USA). Bands were quantified by densitometry using Alphaview version 3.2.2 (ProteinSimple).

### Statistics

Data were analyzed using SPSS Version 20 (Chicago, IL, USA). Initial parturition rates were analyzed across dams using a chi-square analysis (treatment × outcome). For repeated measures data of weight and length across development, linear mixed effects models were used with each pup as the analysis unit nested within litters. This approach allows for multiple mice to be tested within a litter while controlling for litter effects.^37, 38^ Similarly for the social approach task, chamber, treatment, sex and relevant interactions were treated as fixed effects, and dam was treated as a random effect. For all univariate analyses, linear mixed model was used with each experimental offspring as the analysis unit, treatment, sex and treatment by sex interactions as fixed effects, and dam as a random effect to control for litter effects. Immunoblot data were analyzed using a two-way factorial analysis of variance with treatment and sex as independent variables. SERT levels were correlated with behavioral measures using Spearman's rho when applicable. All statistical tests were two-tailed with alpha set at 0.05.

## Results

### Parturition, growth and development

Repeated challenge with aerosolized OVA produced a robust inflammatory response, observed in maternal sera and bronchoalveolar lavage fluid (BALF), with the characteristic allergy/asthma cytokine profile previously described in mice^39, 40, 41, 42^ (see Supplementary Materials). To determine whether this OVA-induced immune activation during pregnancy resulted in altered parturition rates, a chi-square analysis was performed between MAA and PBS-exposed dams for the number of full-term, aborted and cannibalized litters. Out of 27 initial litters, only two litters were cannibalized following parturition (one PBS and one MAA) and two additional pregnancies did not come to term (two PBS, zero MAA). The majority of pregnancies (11 PBS and 12 MAA) were full term with no significant differences between treatment groups, *χ*^2^_(2,*n*=27)_=2.074, *P*=0.355.

**Figure 1:**
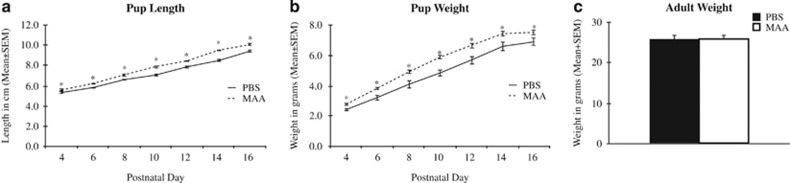
Developmental growth trajectories of offspring born to PBS and MAA-exposed dams. (**a**) Pups born to mothers exposed to MAA were significantly longer throughout juvenile development compared with typically developing control mice. (**b**) Similarly, offspring of MAA dams weighed significantly more than control pups throughout development. (**c**) Differences in weight between treatment groups were no longer present by adulthood at 10 weeks of age. ^*^P<0.05. MAA, maternal allergic asthma; PBS, phosphate-buffered saline.

Offspring born to mothers with repeated exposure to allergic asthma treatment (that is, MAA group) showed altered weight and length developmental trajectories throughout juvenile development (Figure 1). Offspring of both treatment groups showed significant increases in body length as a main effect of developmental day, F_(6,74)_=325.434, *P*<0.001; however, offspring of MAA-exposed dams were significantly longer throughout all developmental time points, F_(1,213)_=79.054, *P*<0.001. Moreover, repeated measures analysis of variance revealed a significant day × treatment interaction for pup length, F_(6,74)_=2.286, *P*=0.044 (Figure 1a). Similarly, although offspring of both MAA and PBS-exposed dams exhibited significant weight gain throughout the initial three weeks of life, F_(6,64)_=216.012, *P*=0.001, offspring of MAA-exposed dams weighed significantly more than PBS controls as indicated by a main effect for treatment, F_(1,198)_=45.992, *P*=0.0001 (Figure 1b). This weight gain was independent of developmental day, F_(6,64)_=1.165, *P*=0.336. Interestingly, by 10 weeks of age, these differences in body mass were no longer present between treatment groups, F_(1,27)_=0.110, *P*=0.742 (Figure 1c).

### Social behaviors

**Figure 2:**
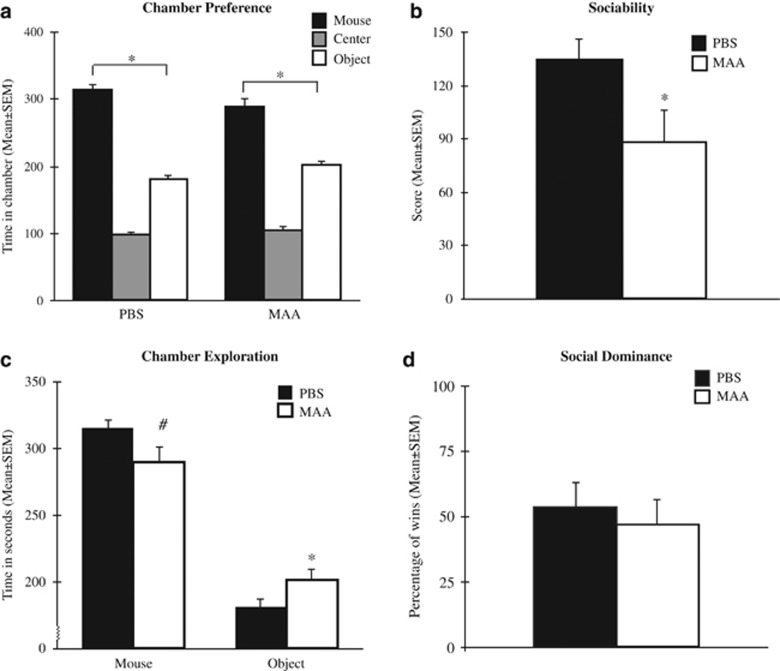
MAA results in social behavior deficits in offspring. (**a**) Offspring of both MAA and control dams showed significant chamber preference for the novel mouse, although the magnitude of preference was reduced in MAA offspring. (**b**) Mice born to mothers repeatedly exposed to allergy/asthma displayed significantly reduced sociability scores compared with control mice from PBS-treated dams. (**c**) Social behavior deficits in MAA offspring were a result of reductions in the total time spent exploring the chamber containing the novel mouse and increases in total time spent exploring the chamber with the novel object. (**d**) On average, mice from both treatment groups won half of all tube test challenges, indicating no differences in social dominance between groups. ^*^P<0.05, ^#^*P*=0.06. MAA, maternal allergic asthma; PBS, phosphate-buffered saline.

During the initial habituation phase of the social approach task, there was no baseline preference for either empty chamber between treatment groups: main effect chamber, F_(1,162)_=0.002, *P*=0.969; main effect treatment, F_(1,162)_=0.036, *P*=0.850; chamber by treatment interaction, F_(1,162)_=0.381, *P*=0.538, indicating no inherent bias at the start of the behavioral task. During the subsequent social approach phase, there was a significant main effect for chamber preference, with mice spending significantly more time in the mouse chamber compared with the object chamber F_(1,162)_=65.979, *P*=0.0001 (Figure 2a). This preference for the social chamber was apparent in both treatment groups as indicated by a null effect for treatment, F_(1,162)_=0.272, *P*=0.603 and treatment by chamber interaction F_(1,162)_=0.606, *P*=0.438. Specifically, both MAA and PBS offspring spent significantly more time in the chamber with the mouse compared with the chamber with the novel object. However, analysis of the strength of the social preference between treatment groups revealed a significant decrease in sociability in MAA offspring compared with PBS control offspring F_(1,79)_=4.225, *P*=0.043. Offspring of MAA-exposed dams had a 33% decrease in the sociability score, indicating a deficit in social behavior compared with offspring of PBS-exposed dams (Figure 2b). This difference in sociability was independent of sex, main effect: F_(1,79)_=0.421, *P*=0.518; sex by treatment interaction: F_(1,79)_=0.836, *P*=0.363. Deficits in sociability between MAA and PBS groups were brought on by a significant increase in the time spent with the novel object by offspring of MAA dams, F_(1,79)_=4.079, *P*=0.047, as well as a trend towards a decrease in the time spent with the novel mouse, F_(1,79)_=3.590, *P*=0.062 (Figure 2c).

No significant differences were observed in social dominance between PBS and MAA groups (Figure 2d). Analysis of variance revealed no difference in the percentage of matches won between treatment groups F_(1,26)_=0.709, *P*=0.407. In fact, offspring from both MAA-exposed dams and control PBS-exposed dams won ~50% of all tube test bouts. The average number of wins was independent of sex as revealed by a null effect for sex, F_(1,26)_=0.061, *P*=0.806, as well as no sex by treatment interaction, F_(1,26)_=0.061, *P*=0.806.

### Repetitive and perseverative behaviors

**Figure 3:**
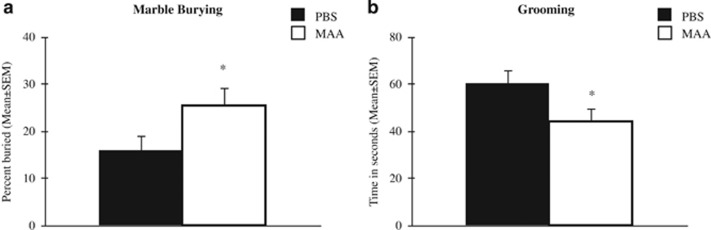
Alterations in stereotypical restricted/repetitive behaviors. (**a**) Offspring of MAA dams buried significantly more marbles than control mice. (**b**) There was a significant reduction in the total time spent grooming observed in mice born to mothers repeatedly challenged with OVA throughout pregnancy. MAA, maternal allergic asthma; PBS, phosphate-buffered saline.

In mice, marble-burying is used as an index of perseverative digging behavior analogous to the restricted, repetitive patterns of behavior observed in neurodevelopmental disorders.^43^ Offspring of MAA-exposed dams buried significantly more marbles compared with offspring of PBS control dams (Figure 3a), as indicated by a main effect for treatment F_(1,78)_=4.854, *P*=0.031. Interestingly, a significant main effect for sex was observed, F_(1,78)_=6.340, *P*=0.014, with male mice burying a greater percentage of marbles compared with females. This sex difference was independent of treatment as noted by a null sex by treatment interaction: F_(1,78)_=2.074, *P*=0.154.

The total time spent grooming during a 10-min period was significantly reduced in offspring of MAA-exposed dams compared with typically developing mice born to control dams (Figure 3b). When controlling for variations across litters, linear mixed effects model revealed a significant main effect of treatment for grooming times F_(1,81)_=4.21, *P*=0.043. On average, there was a 25% reduction in grooming time for offspring of MAA-exposed dams (mean=44.36, s.d.=34.30) compared with offspring of PBS-exposed dams (mean=60.41, s.d.=36.49). Interestingly, there was a significant main effect for sex, F_(1,52)_=5.380, *P*=0.024, with males spending significantly more time grooming compared with females. This sex-specific difference was apparent regardless of treatment group as observed by a null sex by treatment interaction, F_(1,52)_=0.038, *P*=0.846.

### Hypo/hyperactivity

**Figure 4:**
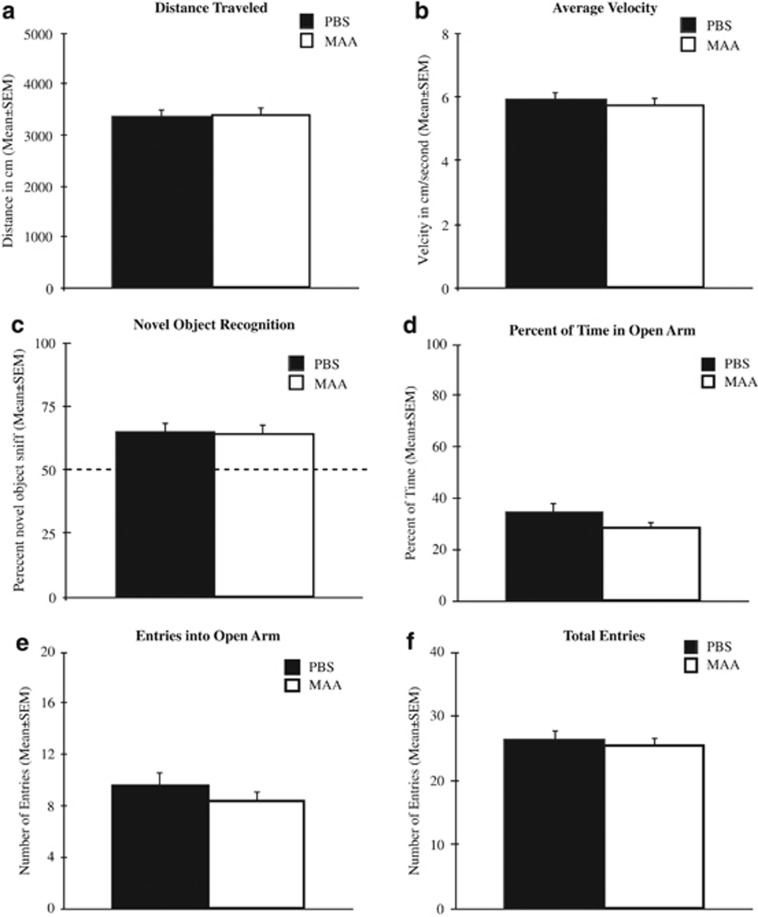
Measures of locomotor activity, memory formation and anxiety. No differences were observed in (**a**) the total distance traveled or (**b**) average velocity between treatment groups during the open-field observations. (**c**) Mice from both MAA- and PBS-exposed dams showed normal object recognition as measured by a preference (that is, >50% sniffing) for a novel versus familiar object. (**d**) There were no differences observed in the percent of time spent in the open arms of the elevated plus maze arena between treatment groups. (**e**) Similarly, no differences were observed in the total number of entries into the open arm between offspring of PBS-treated and MAA dams. (**f**) These similarities parallel similarities in the total number of entries into all arms of the maze, an internal control for locomotor activity. MAA, maternal allergic asthma; PBS, phosphate-buffered saline.

To determine whether phenotypic differences between offspring of MAA- and PBS-exposed dams were due to hyper/hypoactivity, mice were placed in an open arena and measured for locomotor behavior. Between treatment groups, no differences were observed in either the total distance traveled, F_(1,27)_=0.001, *P*=0.994 (Figure 4a), or average velocity, F_(1,27)_=0.165, *P*=0.688, demonstrating similar locomotor activity between offspring of MAA- and PBS-exposed dams (Figure 4b). Moreover, there were no main effects for sex (distance traveled, F_(1,27)_=0.538, *P*=0.496; average velocity, F_(1,27)_=0.317, *P*=0.578) and no sex by treatment interactions (distance traveled, F_(1,27)_=0.037, *P*=0.849; average velocity, F_(1,27)_=0.004, *P*=0.849).

### Learning and memory

To determine whether reductions in social behavior are due to deficits in cognition, offspring from MAA- and PBS-exposed dams were measured for alterations in memory performance using the novel-object recognition task. One day following the memory acquisition phase, offspring of MAA- and PBS-exposed dams spent significantly more time exploring the novel object, as indicated by a novel-object exploration time >50% (one-sample *t*-test): PBS, *t*(15)=3.569, *P*=0.003; MAA, *t*(15)=3.967, *P*=0.001. There were no significant differences in the percentage of time spent exploring the novel object between sex, F_(1,27)_=1.608, *P*=0.216 or treatment groups, F_(1,27)_=0.003, *P*=0.954, suggesting equal memory formation between offspring of PBS and MAA dams (Figure 4c).

### Anxiety-like behavior

There was no significant difference in the percentage of time spent in the open arms of the elevated plus maze between treatment groups, F_(1,49)_=1.789, *P*=0.187, as well as no difference between sex F_(1,49)_=0.018, *P*=0.892 or sex by treatment interactions F_(1,49)_=0.182, *P*=0.671, indicating an absence of a high anxiety-like phenotype in offspring of MAA-exposed dams (Figure 4d). Moreover, offspring of both MAA and PBS-exposed dams made equal number of entries into the open arms of the maze F_(1,49)_=0.796, *P*=0.377 (Figure 4e) as well as similar number of total entries into any arm F_(1,49)_=0.589, *P*=0.447 (Figure 4f).

### Serotonin transporter expression

**Figure 5:**
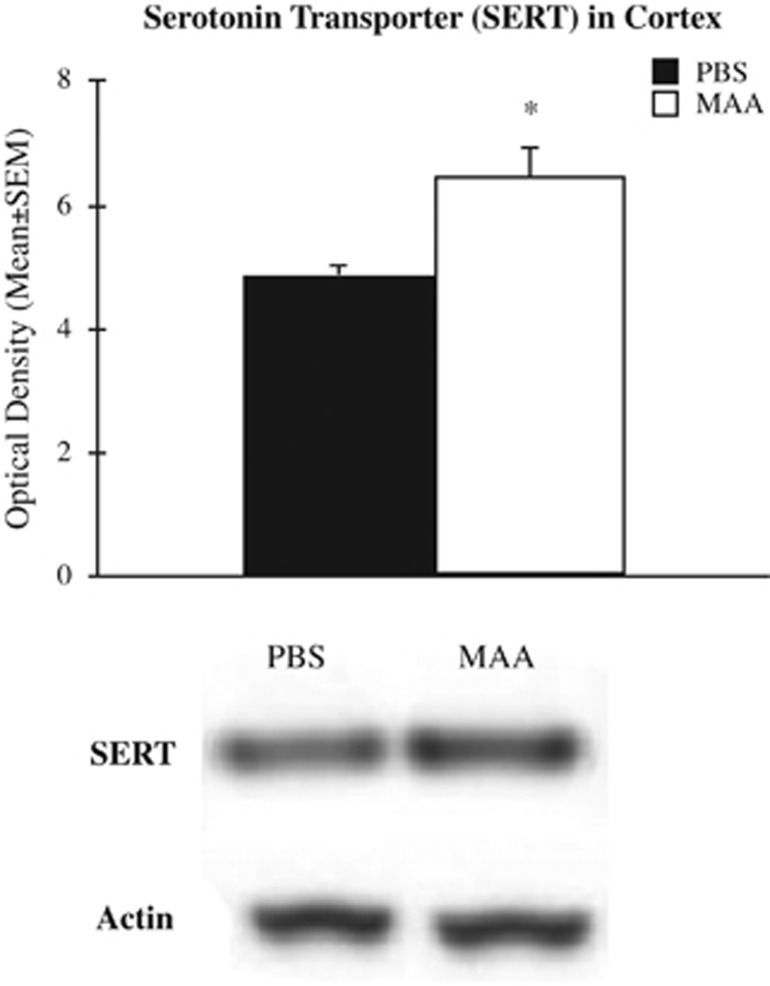
Immunoblot analysis of serotonin transporter (SERT) protein expression in the cortex. Mice born from mothers sensitized to OVA before pregnancy and repeatedly challenged with aerosolized OVA during pregnancy had significantly greater expression of SERT in the cortex compared with typically developing mice born to PBS control dams. ^*^P<0.05. MAA, maternal allergic asthma; OVA, ovalbumin; PBS, phosphate-buffered saline.

The analysis of serotonin transporter protein levels in the cortex of MAA and PBS offspring revealed a significant main effect for treatment, F_(1,12)_=9.67, *P*=0.011. Mice born to dams exposed to MAA had a 33% increase in the expression of SERT compared with control offspring of PBS-exposed dams (Figure 5). These differences did not differ between males and females as indicated by a null effect for sex, F_(1,12)_=0.678, *P*=0.429, as well as no sex by treatment interactions, F_(1,12)_=0.066, *P*=0.803. Increases in SERT expression in the cortex positively correlated with marble-burying behavior, rs(14)=0.602, *P*=0.023, with mice expressing the highest SERT levels burying the greatest number of marbles. These correlations between SERT expression and behavior were not evident in any other behavior measured.

## Discussion

Activation of the maternal immune system with an allergy/asthma insult significantly perturbed developmental growth and species-typical behaviors in offspring. Our maternal allergy/asthma model produced an offspring phenotype characteristic of impaired social approach. Animal models of maternal immune activation using the viral mimic polyI:C report similar reductions, but not elimination, of sociability.^21, 33^ Offspring of MAA dams also exhibited increased marble-burying behavior, a perseverative behavior analogous to repetitive behaviors observed in neuropsychiatric disorders including obsessive compulsive disorder and ASD.^43^ In addition, we observed disturbances in the serotonin pathway with increased SERT evident in offspring of MAA-exposed dams. Excessive marble burying positively correlated with SERT levels and may reflect hyperactivity or motor stereotypies that are reminiscent in ADHD and ASD. Surprisingly, offspring of MAA-exposed dams exhibited decreased self-grooming, a behavioral trait often used to model the repetitive self-directed behaviors in individuals with ASD. Given that others have also noted incongruence between grooming and marble-burying behavior in mice,^44, 45^ the discrepancies in these behavioral responses suggests the presence of diverse underlying mechanisms for different types of repetitive behaviors and underscore the importance of identifying endophenotypes that may be fundamental to behaviors observed across different neurodevelopmental disorders.

The sociability deficits observed in MAA offspring model the social behavior impairments characteristic of many neurodevelopmental disorders. These impairments are hypothesized to be secondary expressions of more fundamental deficits in brain processes related to attention, motivation and neurospecialization for social cognition.^46, 47^ Although initial measures of basic learning/memory processes using the novel-object recognition task appear to be intact, more specialized cognitive functions are warranted to tease apart endophenotypes associated with maternal allergy and asthma responses. Indeed, novel-object recognition provides a rapid measure of memory formation and learning but is limited in its specificity for assessing attention.^48^ More precise attention tasks requiring sustained attention such as the five-choice serial reaction time test may provide a deeper understanding of the neural alterations imparted by MAA. Similarly, other fundamental brain processes including changes in motivation and social recognition may be contributing to the social behavior deficits observed. For example, in a strain-specific mouse model of ASD, the BTBR mouse exhibits complex cognitive deficits in probabilistic learning, attention, social motivation and transitive inferences that likely underlie the social behavior deficits characteristic of this mouse model.^49^ Thus, further exploration of how MAA alters more fundamental processes contributing to social behaviors is needed to identify the distinct mechanisms contributing to reduced social approach.

Aside from the behavioral phenotype observed in offspring of MAA dams, repeated OVA challenges throughout gestation resulted in altered weight and length developmental trajectories. That is, offspring born to MAA-exposed dams were significantly longer and heavier throughout juvenile development compared with control offspring. Interestingly, a similar developmental pattern was observed in offspring of mice exposed to human IgG antibodies of mothers with a child diagnosed with autism.^50^ Prenatal exposure to environmental insults, including factors contributing to allergies and asthma, are associated with childhood obesity,^51^ a condition that includes both systemic and neuro-inflammation.^52^ Increased weight gain in offspring of MAA dams may represent priming and reprogramming of metabolic function through both immune and nervous system mechanisms, as has been previously hypothesized.^53, 54^ In fact, a recent examination of metabolic changes in immune cells of mice born to dams exposed to a viral mimic revealed persistent mitochondrial dysfunction in splenocytes.^55, 56^ Interestingly, comorbid metabolic dysregulation is highly prevalent in neurodevelopmental disorders including ASD,^57^ ADHD^58^ and schizophrenia,^59^ suggesting that environmental insults may impart pervasive changes in homeostasis and regulation across organ systems.

The behavioral and physiological changes observed in offspring of MAA-exposed dams during gestation strongly suggest that maternal allergy/asthma alters basic developmental processes that underlie fetal development. The serotonin pathway may link increased immune activation during gestation with changes in neural function in the offspring. It has previously been demonstrated that peripheral immune responses can lead to changes in serotonin levels in various brain regions.^26^ Numerous cytokines have been shown to alter brain serotonin signaling through disruption in transmitter synthesis and up/down regulation of serotonin receptor expression.^60, 61, 62^ For example, interleukin-4 (IL-4), a cytokine highly expressed in allergy/asthma responses and observed in BALF and sera of MAA dams, dose-dependently alters serotonin uptake through modulation of SERT function.^60^ Consequently in the current study, mice born to dams repeatedly challenged with OVA during gestation had increased levels of SERT protein in the cortex compared with offspring of dams exposed to PBS during gestation. These increases in SERT expression positively correlated with repetitive marble-burying behavior. Interestingly, drugs that block SERT function, such as selective serotonin reuptake inhibitors, suppress the marble-burying behavior in mice, further supporting the link between serotonin reuptake and repetitive behaviors.^63^ The SERT protein is important for regulating synaptic serotonin levels and alterations in serotonin function are implicated in a host of neuropsychiatric diseases,^64^ including the repetitive stereotyped behaviors characteristic of ASD.^65^ Disruptions in serotonin signaling, particularly through gene-coding variants in SERT, are implicated in numerous neurodevelopmental disorders.^66, 67, 68^ Increases in SERT levels in the offspring of MAA dams may result in excessive uptake of serotonin and a reduction in postsynaptic signaling. In mice, gain-of-function coding variants for the human SERT gene (*SLC6A4*) results in altered homeostasis of serotonin levels and behavioral deficits.^69^ Moreover, in a rat model of maternal immune activation fetal brains of dams exposed to poly(I:C) exhibited increased serotonergic neurons and reductions in serotonin content.^70^ Considering the role of serotonin in behavior, the sensitivity of the developing serotonin system to perturbations by immune system signaling, and the links between SERT gene variants and neurodevelopmental disorders, the serotonin system is a likely target where genetic and environmental insults converge to disrupt neurodevelopment.

For almost two decades, researchers have relied on a classic experimental model of allergic asthma in which mice are systemically sensitized to a foreign antigen (that is, OVA) to induce the production of OVA-specific T_H_2 cells and OVA-specific IgE antibodies.^71^ Subsequent exposures to aerosolized OVA results in rapid mass infiltration of T_H_2 cells in the airways, increased mucus production and development of airway hyper-responsiveness. This model produces the hallmark immunological responses analogous to those observed in humans including elevated levels of the cytokine profile (for example, IL-4 and IL-5) governing the initiation and progression of asthma.^71, 72^ Traditionally, the Balb/c mouse has been the most widely used model to study allergic asthma as this strain has a prototypical T_H_2-skewed phenotype.^39, 73^ However, the use of this mouse strain in our experimental model is confounded as Balb/c mice exhibit innate deficits in social approach and increased anxiety,^74^ thus limiting its use in the study of the behavioral consequences of MAA. Conversely, although the highly social C57Bl/6 strain has previously been used in the OVA-induced asthma paradigm, it possesses a T_H_1 polarized phenotype resulting in an attenuated allergic response when compared with the Balb/c mouse.^41^ Importantly, this predisposition does not prevent an allergic phenotype from developing upon OVA sensitization and subsequent OVA challenge but may be protective in mitigating its full behavioral effects. The genetic predisposition for T_H_1- or T_H_2-specific polarization coupled with their associated behavioral phenotypes provide additional insight into possible connections between immune activation and behavioral and biological responses in general and also specific to allergies and asthma. That is, the low sociability inherent in the Balb/c strain may be, in part, due to its underlying T_H_2-skewed immune profile. These observations from our model and from the Balb/c strain eloquently demonstrate the important contributions of both genes and environment on neurodevelopment and highlight the importance of strain selection when measuring the neuroimmunological effects of discrete environmental insults.

To date, there are few epidemiological studies examining the neurodevelopmental risk factors associated with maternal allergies and asthma. A recent report exploring the maternal cytokine profiles that may act as risk factors contributing to neurodevelopmental disorders identified an altered cytokine profile during the second trimester in women who gave birth to a child later diagnosed with ASD.^6^ This cytokine profile showed elevated IFN-γ, IL-4 and IL-5 and was considered consistent with an allergic/asthma clinical phenotype in humans,^75, 76, 77^ suggesting that allergy/asthma exposure may be a factor that drives maternal immune activation and increased risk for ASD. Moreover, Goines *et al.*^6^ note that a different maternal cytokine profile during pregnancy consisting of elevated IL-6 and IL-2 were associated with having a child with other developmental disorders, but not ASD. In our MAA model, we observed increased IL-4 and IL-5 in both sera and BALF in mice challenged with OVA during pregnancy. Similarly in humans, amniotic fluid taken from mothers whose children were later diagnosed with ASD had elevated levels of IL-4, whereas those with other developmental neuropsychiatric disorders were noted to have increased levels of IL-6.^78^ Importantly, IL-6 is a pro-inflammatory cytokine released in response to a range of immune-stimulating agents, including both viral/bacterial infections and allergy/asthma, and is linked to a host of neurological abnormalities and cognitive impairments not specific to ASD.^79, 80, 81^ A large epidemiological study demonstrated that mothers with new-onset allergies and asthma during the time of pregnancy were at increased risk for having a child with ASD.^17^ Interestingly, this is not consistent with another study that looked at maternal allergies and asthma irrespective of whether they were new onset or existing conditions and found no association with ASD.^18^ The differences between studies may be partly due to difference in ongoing treatment for asthma/allergies and the suppression of immune activation by individuals with an ongoing history of allergies/asthma. This variability in the clinical population suggests that strategies may be present to mitigate the neurodevelopmental consequences of maternal allergies/asthma and imparts a need to explore how various treatments commonly employed in allergy and asthma response may also alter fetal development.

## Conclusions

Allergy/asthma-induced activation of the maternal immune system imparts behavioral and neurobiological alterations in offspring that resemble features observed across neurodevelopmental disorders. These findings are concerning given the range of stimuli associated with allergies and asthma. For example, exposure to air pollutants, such as diesel fuel and other particulate matter, can result in the development of asthma, and exposure to pollutants is associated with an increased risk of having a child with a neurodevelopmental disorder.^82, 83^ Similarly, phthalates and other plasticizers ubiquitous in our environment are known neurotoxins associated with both increased risk of asthma and neurodevelopmental disorders,^84, 85, 86, 87^ highlighting the myriad environmental triggers that may be contributing to the etiology of these diseases. Together, the behavioral consequences of maternal allergy/asthma along with the observed changes in brain chemistry support the notion that the maternal immune system has a crucial role in shaping fetal development and is an important factor contributing to the etiology of neurodevelopmental disorders.
