# Supplementary material for: Allergic fetal priming leads to developmental, behavioral and neurobiological changes in mice
Source: Transl Psychiatry. 2015 Apr 7;5(4):e543–. doi: 10.1038/tp.2015.40 (PMC4462603; doi:10.1038/tp.2015.40)
Supplement: Supplementary Information [file tp201540x1.pdf]

Supplementary Materials: Quantification of cytokine and chemokine levels in maternal **(A)** Bronchoalveolar Lavage Fluid (BALF) and **(B)** Sera. On gestational day 17.5, maternal BALF and sera were collected four hours following the final challenge of aerosolized OVA or PBS and supernatants were analyzed using multiplexing bead immunoassays (Millipore, Bellerica, MA, USA). Briefly BALF was collected by flushing the lungs with 1 mL of PBS a total of four times. The resultant BALF was pooled and frozen until analysis. Maternal blood was collected intracardially and maintained at room temperature for at least 30 minutes to clot. Blood was then spun at 5,000 rpm and the sera was collected and frozen for later analysis. Cytokine analysis was performed as recommended by the manufacturer's specifications. Briefly, 25ml of supernatant was incubated with antibody-coupled beads. After a series of washes, a biotinylated detection antibody was added to the beads, and the reaction mixture was detected by the addition of streptavidin–phycoerythrin. The bead sets were analyzed using a flow-based Luminex 100 suspension array system (Bio-Plex 200; Bio-Rad Labora- tories, Hercules, CA, USA). Unknown sample cytokine concentrations were calculated by Bio-Plex Manager software using a standard curve derived from the known reference cytokine concentrations supplied by the manufacturer. A five-parameter model was used to calculate final concentrations and values are expressed in pg/ml. Concentrations obtained below the limit of detection were assigned a value of one-half the limit of detection for statistical comparisons. Supernatant aliquots had not undergone any previous freeze/thaw cycle. Mann-Whitey U exact p-values (one-tailed), $*p < 0.05$

Cytokine	Broncholveolar Lavage Fluid (BALF) Median (pg/ml)		Maternal Sera Median (pg/ml)	
	PBS	*MAA*	*PBS*	*MAA*
IL-1β	2.70	2.19	6.01	14.17
IL-2	2.98	3.57	2.32	3.89
IL-4	**0.20**	**9.34***	**0.55**	**2.21***
IL-5	**0.50**	**14.14***	**5.33**	**64.22***
IL-6	**0.55**	**94.19***	0.55	6.6
IL-7	0.78	2.31	12.36	12.9
IL-10	1.0	3.78	**1.00**	**8.74***
IL-12(p40)	1.95	1.95	1.95	1.95
IL-13	2.4	4.15	2.40	84.39
IP-10	**5.67**	**93.325***	**207.79**	**265.66***
MIP-1α	**3.85**	**20.49***	3.85	28.29
TNFα	**1.15**	**10.48***	3.57	6.36
